# Multi-modulated frequency domain high density diffuse optical tomography

**DOI:** 10.1364/BOE.467614

**Published:** 2022-09-13

**Authors:** Guy A. Perkins, Adam T. Eggebrecht, Hamid Dehghani

**Affiliations:** 1 University of Birmingham, Sci-Phy-4-Health Centre for Doctoral Training, College of Engineering and Physical Sciences, Birmingham, B15 2TT, UK; 2 University of Birmingham, School of Computer Science, College of Engineering and Physical Sciences, Birmingham, B15 2TT, UK; 3 Mallinckrodt Institute of Radiology, Washington University School of Medicine, St Louis, Missouri, 63110, USA

## Abstract

Frequency domain (FD) high density diffuse optical tomography (HD-DOT) utilising varying or combined modulation frequencies (mFD) has shown to theoretically improve the imaging accuracy as compared to conventional continuous wave (CW) measurements. Using intensity and phase data from a solid inhomogeneous phantom (NEUROPT) with three insertable rods containing different contrast anomalies, at modulation frequencies of 78 MHz, 141 MHz and 203 MHz, HD-DOT is applied and quantitatively evaluated, showing that mFD outperforms FD and CW for both absolute (iterative) and temporal (linear) tomographic imaging. The localization error (LOCA), full width half maximum (FWHM) and effective resolution (ERES) were evaluated. Across all rods, the LOCA of mFD was 61.3% better than FD and 106.1% better than CW. For FWHM, CW was 6.0% better than FD and mFD and for ERES, mFD was 1.20% better than FD and 9.83% better than CW. Using mFD data is shown to minimize the effect of inherently noisier FD phase data whilst maximising its strengths through improved contrast.

## 1. Introduction

Near infrared spectroscopy (NIRS) is a growing field in medical imaging that is used in various applications ranging from psychology [1] to medicine [2] and physiology [3]. NIRS can provide pathophysiological information, such as characterising breast tumours [4], however the more common use of NIRS is within neuroimaging [5], known as functional NIRS (fNIRS). fNIRS uses near infrared light which can be emitted and detected as defined by three different paradigms, continuous wave (CW), frequency domain (FD) and time domain (TD). CW is when light is emitted and only the attenuation of light that travels through the medium is detected at the surface is recorded. TD utilises a pulse of light, typically in 10s of picoseconds in width, is emitted and the time of flight is recorded, that is used to build up a distribution of time of flights of photons. The relative merits and optical information obtained by these differing paradigms are described by Scholkmann et al. [6] (CW), Fantini et al. [7] (FD) and Yamada et al. [8] (TD).

This study focuses on FD measurements, where the amplitude of light is sinusoidally modulated at a modulation frequency, *f* (MHz) and the light measured that has been transmitted through a medium at that same modulation frequency *f* is detected, but with a phase-shift as governed by the average photon path. This means the signal has three components, the DC intensity, which is analogous to CW measurement, the AC intensity (I) , which is the intensity of light oscillating at the given modulation frequency, and the phase ( ϕ ), which represents the phase difference in degrees of the emitted and detected modulated light. The phase of the detected light therefore provides information regarding how much it has been delayed (on average) as a result of photon interactions with a medium, i.e scattering. The phase is directly related to the time delay of light, τ , such that ϕ is in the order of ωτ , where ω=2πf and ω is the angular modulation frequency. For a good signal to noise ratio, it is required that ωτ≈1(radians) [9,10]. If ωτ was ≪ 1, there would not be a measurable phase change. If ωτ was ≫ 1, then the AC amplitude decreases and could be below the level of noise.

In the context of imaging biological tissue, time delays are on the order of nanoseconds (for source-detector separations of 2-3 centimetres), which yields modulation frequencies in the order of 100 MHz. For a source-detector separation of r, the measured signal is given by Eq. (1) [7],

(1) $$Signal=DC(r)+AC(r,\omega )e^{i\phi (r,\omega )}e^{-i\omega t}\;,$$

where the DC(r), AC(r, ω ) and ϕ(r,ω) denote the distance and modulation frequency dependencies of three measurable components of the signal. For the case that ω=0 MHz, Eq. (1) yields the CW case, which is just the DC amplitude, as it is shown in equations 12.3-12.5 in [10] that AC(r, ω = 0) = DC(r) and ϕ (r, ω = 0) = 0.

From these measurements, changes in intensity and phase can be recorded, which are then used to obtain changes optical properties, namely absorption ( $\mu_{a}$ ) and reduced scattering ( $\mu_{s}^{\prime }$ ). In biological tissue imaging, these are then used to recover changes in chromophore concentration, which are typically oxygenated haemoglobin (HbO) and deoxygenated haemoglobin (HbR), but can also include cytochrome c oxidase, lipid and water for sampling at 3 or more wavelengths. Spatially overlapping measurements may be used together to perform tomographic reconstructions, which is called diffuse optical tomography (DOT). Since the mid 2000’s [11,12], DOT has advanced by using a ’high density’ (HD) array of sources and detectors. HD-DOT is known to increase the imaging performance of DOT, particularly in localization and image resolution [13].

In the context of FD-HD-DOT, it has been shown on human head model simulations that the use of phase data increases imaging performance as compared to intensity (CW-HD-DOT) measurements [14]. That study demonstrated phase data sampled deeper into tissue than intensity alone and was less sensitive to superficial tissue contamination, showing that phase data should be used in FD-HD-DOT.

### 1.1 Multi modulation frequency studies

As described in Eq. (1), the signal obtained in FD measurements is dependent on the modulation frequency used. The current literature on the use of varying modulation frequencies can be categorised into three; simulation, data collected on a phantom and data collected in-vivo. Some studies have evaluated the use of a single modulation frequency at a time, whereas some combine and use multiple modulation frequencies simultaneously in image reconstruction, which will be referred to as multi-frequency FD (mFD). A summary of these studies can be found in Table 1 and will be outlined.

**Table 1.** A table containing a summary of research within mFD measurements. The studies are ordered and categorized into simulations (Sim.), data collected on a phantom (Phant.) and then data collected in-vivo. Within the categories, studies are ordered by year from oldest to most recent. The geometry of the measurement is summarized, the range and number of wavelengths used ( n/a means a single value of $\mu_{a}$ , $\mu_{s}^{\prime }$ was used, λ(N) ), as well as the range of modulation frequencies and number of frequencies (N) used in image reconstruction at a time. Then if the study reports absorption and/or scattering parameters and finally the type of output of the reconstructed data.

Ref	Data	Geometry	λ /nm (N)	ω / MHz (N)	$\Delta \mu_{a}/\mu_{s}^{\prime }$	Output
[17]	Sim.	Slab	n/a	20-500 (7)	Both	2D DOT
[21]	Sim.	Circle	n/a	100-250 (4)	Both	HD-DOT
[25]	Sim.	Head	n/a	0-600 (2)	$\Delta \mu_{a}$	3D HD-DOT
[26]	Sim.	Head	690-850 (2)	0-1000 (1)	$\Delta \mu_{a}$	3D HD-DOT
[22]	Sim.	Circle	n/a	100-200 (3)	Both	2D HD-DOT
[24]	Sim.	Cuboid	n/a	78-203 (3)	$\Delta \mu_{a}$	3D HD-DOT
[23]	Sim.	Circle	n/a	100-1000 (5)	Both	2D HD-DOT
[15]	Phant.	Cylinder	755 (1)	10-1000 (1)	Both	2D NIRS
[16]	Phant.	Cylinder	674-956 (4)	0-1000 (1)	Both	2D NIRS
[18]	Phant.	Cylinder	665-830 (4)	110-280 (6)	Both	3D DOT
[19]	Phant.	Cylinder	665-830 (4)	110-280 (6)	Both	3D DOT
[27]	Phant.	Cylinder	690-980 (6)	50-300 (35)	Both	2D HD-DOT
[27]	In-Vivo	Arm	689-850 (2)	50-300 (35)	Both	2D NIRS
[20]	In-Vivo	Cervix	690-970 (7)	130-490 (36)	Both	2D NIRS

The use of different modulation frequencies in FD measurements was first reported by Patterson et al. [15] in 1991, they used single modulation frequencies across 10 MHz to 1000 MHz to spectroscopically recover the absorption and reduced scattering of a tissue mimicking phantom and building on this, similar work was done by Pham et al. [16] in 2000. Then in 2005 Intes et al. [17] simulated the use of mFD-DOT (2D), using up to 13 simultaneous modulation frequencies from 20 MHz to 500 MHz in 40 MHz increments. In their results, they found that using more than 7 frequencies yielded little additional benefit and modulation frequencies combined too close to each other would contain redundant information. The following year in 2006, Burcin Unlu et al. [18] performed mFD DOT on a cylindrical phantom using frequencies from 110 MHz to 280 MHz in 30 MHz increments. The higher number of combinations (total of 6) performed the best, followed by a combination of 3 frequencies (110 MHz + 200 MHz + 260 MHz), then 2 (110 MHz + 260 MHz) and then 200 MHz alone. Also in 2006, Gulsen et al. [19] used spectral (665 nm, 785 nm , 808 nm and 830 nm) mFD-DOT measurements on the same phantom to perform similar mFD-DOT analysis.

The first use of clinical mFD measurements was in 2010 by Hornung et al. [20], where they used mFD measurements on 13 patients, imaging the uterine cervix during regular pregnancies to obtain physiological information (HbO, HbR and oxygen tissue saturation). To date, this appears to be the only use of mFD measurements in a clinical setting and no studies have used mFD for in-vivo human brain imaging.

Since 2010, only one mFD study has included real data and the rest have been simulations. In 2015, Chen et al. [21] simulated the first use of HD-DOT on a 2D circular model, using frequencies between 100 MHz to 250 MHz in 50 MHz increments, as well as a new clustered sparsity reconstruction method to reconstruct absorption and reduced scattering coefficients simultaneously. They used 15 combinations of frequencies and found that the best imaging performance was from the combination of all four modulation frequencies. Then in 2021, Mudeng et al. [22] and Shifa et al. [23] also simulated HD-DOT on a 2D circular model, using 3 frequencies, 100 MHz, 150 MHz and 200 MHz and found that mFD measurements of 100 MHz + 150 MHz and 100 MHz + 150 MHz + 200 MHz performed similarly to that of 200 MHz alone. Shifa et al. used frequencies between 100 MHz to 1000 MHz, however they only analysed up to 700 MHz. They found that the best tomographic reconstructions came from the combination of 100 MHz increments from 200 MHz to 700 MHz, however they assumed the same level of noise (20dB) for all modulation frequencies, which is a limitation of the study, as there is no penalty for the additional information content.

Perkins et al. [24] in 2021 was the first study to simulate mFD-HD-DOT on a 3D model, using a cuboid 3 layered (skin/scalp, gray matter, white matter) and frequencies of 78 MHz, 141 MHz and 203 MHz, as available from a commercial system, the ISS Imagent. Focal activation were simulated and the use of intensity v.s phase v.s intensity & phase combined data, at the three modulation frequencies were compared. To evaluate the relative performances, the localization error of activations, full width half maximum error and effective resolution were used as metrics [13]. The most accurate reconstructions of absorption coefficient came from when intensity data at 141 MHz was combined with phase data at 78 MHz + 141 MHz + 203 MHz, both in the case of no noise and noise-added data. Phase data performed better than intensity in the no noise case, but became worse than intensity in the presence of noise. However, the combination of intensity and phase with noise was better than intensity with noise alone.

The final two simulation studies also in 2021 were from DeJong et al. [25] and Fan et al. [26], where they both use 3D atlas models of the human adult head for HD-DOT, with Fan using 5 different head models. Fan uses 11 frequencies between 0 to 1000 MHz and found that without noise 700 MHz gave the most accurate localization of brain activations and 1000 MHz the lowest full width half maximum error in reconstruction of optical properties. The inclusion of noise and when including nearest neighbour 3 and 4 measurements respectively, 300 MHz performed the best. DeJong uses single value decomposition of the Jacobians (Sensitivity mapping function) of intensity and phase, of single and combined frequencies between 100 MHz to 600 MHz in increments of 100 MHz to evaluate which combinations hold the most information content. Using this analysis and evaluating the localization of brain activation and the full width half maximum error, the combination of 500 MHz + 600 MHz performed the best.

Finally towards the end of 2021 Stillwell et al. [27] created a real time broadband mFD system, capable of NIRS and DOT (a scale-able system) to perform HD-DOT. Their system uses 6 wavelengths between 690 nm and 830 nm with modulation frequencies from 0 to 400 MHz in increments of 0.1 MHz. They performed system characterisation and calibration of the device using a block phantom with a simulated tumour inclusion 1 mm below the surface of 10 mm to 30 mm diameter. Then they took measurements on tissue to perform high speed quantification of hemodyanmics by virtue of 2D spatial topographic mapping. They performed in-vivo arterial occlusion measurements by placing a blood pressure cuff on the upper arm of a healthy volunteer. This was the first demonstration of real time multi wavelength broadband mFD measurements and paves the way for further mFD studies in a research and clinical research setting.

Following from the studies described in Table 1, this study will be the first to use real mFD HD-DOT measurements to reconstruct parameters in 3D. Previous studies have either been single frequency 2D NIRS (Patterson et al. [15] and Pham et al. [16]), or mFD-DOT (Burcin Unlu et al. [18] and Gulsen et al. [19]) and not mFD-HD-DOT. The significant performance increase, and therefore importance of ’high density’ DOT compared to non ’high density’ measurements have been shown [13] and used in-vivo for mapping of distributed brain function [5]. As well as this, Burcin Unlu et al. [18] and Gulsen et al. [19] perform their measurements on circular phantoms, having sources and detectors around the entire perimeter of the phantom. This study will use source and detectors on one surface of a cuboid block phantom, which represents a more realistic (in-vivo human brain imaging) use case.

Quantitative evaluations of how mFD-HD-DOT measurements perform have only been simulated Perkins et al. [24], DeJong et al. [25] and Fan et al. [26]), so evaluating the performance of mFD on a phantom will eliminate factors which can effect the measurement outside of type of data itself, such as poor source-detector coupling and instrumental noise. As well as this, it allows for flexibility in the processing of data and methods for image reconstruction, due to the simple geometry and stable measurements on a phantom. Three modulation frequencies will be used of 78.125 (78) MHz, 140.625 (141) MHz and 203.125 (203) MHz, which are the same as the simulated mFD-HD-DOT evaluation as seen in Perkins et al. [24] . The use of 3 frequencies is more than sufficient to observe the benefits of mFD and the difference between the 3 frequencies is such that there is a sufficient difference in data between the frequencies (Intes et al. [17], Burcin Unlu et al. [18], Chen et al. [21] and Mudeng et al. [22] .).

Whilst the most common approach for image reconstruction in HD-DOT/DOT is a single linear iteration in parameter recovery, this study will also investigate the use of an iterative approach for image reconstruction, so that the relative performance of mFD v.s FD v.s CW can be assessed in both the single iteration and iterative reconstruction case.

## 2. Methods

A mechanically switchable solid inhomogeneous phantom was used for data collection and detailed information about the phantom can be found elsewhere [28]. The bulk properties of the phantom at 830 nm are $\mu_{a}$ = 0.01 ${\mathrm{mm}}^{-1}$ and $\mu_{s}^{\prime }$ = 0.7 ${\mathrm{mm}}^{-1}$ . The phantom consists of a homogeneous 3D geometry, with a cylindrical hole running across it. The center of the hole is at 15 mm from the surface of the phantom and allows a rod to be inserted into it. The rod is inhomogenous as it contains a small cylindrical anomaly, made of black polyvinyl chloride (pvc), which causes it to act as a contrast as compared to the rest of the cylinder, which is made of the same material as the rest of the phantom. The rod can be moved across the phantom, and hence the position of the contrast anomaly can be changed. For reference, the optical properties of contrast anomaly are given in Table 2, these are for a $\mu_{s}^{\prime }$ of approximately 1 ${\mathrm{mm}}^{-1}$ , which holds true around 700 nm.

**Table 2.** The dimensions of the contrast anomalys within each rod used [28]. Rod N has a diameter of N mm and length N mm. The equivalent $\Delta \mu_{a}$ is for a $\mu_{s}^{\prime }$ of approximately 1 ${\mathrm{mm}}^{-1}$ , which holds true around 700 nm and is derived using the equivalence relation [29] and an inclusion of 10 ${\mathrm{mm}}^{3}$ .

Diameter / mm	Length / mm	Volume / ${\mathrm{mm}}^{3}$	Equivalent $\Delta \mu_{a}$ / ${\mathrm{mm}}^{-1}$
3	3	21	0.005
5	5	98	0.017
7	7	269	0.040

**Fig. 1.**
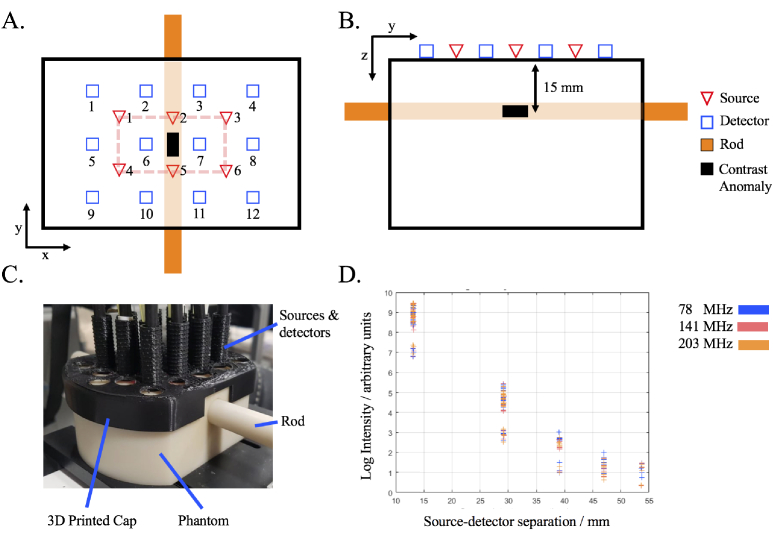
A. (z plane) and B. (x plane) A schematic diagram of the source-detector placement on top of the phantom, as well as the orientation of the movable rod which contains the contrast anomaly. For contrast anomaly measurements, the anomaly is centred at x = y = 0 mm and z = -15 mm. C. A photo of the phantom with the rod inserted. The sources and detectors are placed on the phantom using a 3d printed cap. D. A graph showing the log of the intensity as a function of source-detector separation at three modulation frequencies.

Measurements were taken using the ISS Imagent (ISS, Champaign, IL, USA). Six sources and twelve detectors were placed in a high density array [11,12] on top of the phantom via a 3D printed cap. Each source emits light at 830 nm and 690 nm. The light emitted is sinusoidally modulated and measurements were taken at 78 MHz, 141 MHz and 203 MHz. Measurements were sampled at 39.73 Hz. The array of sources and detectors were centered about the middle of the phantom. This created five nearest neighbour (NN) measurements respectively of 13 mm (NN1), 29 mm (NN2), 39 mm (NN3), 46 mm (NN4) and 53.64 mm (NN5). Only measurements of NN1 (24 channels) and NN2 (28 channels) were used for image reconstruction, as measurements of NN3 to NN5 were deemed to have a too low signal to noise ratio. The source detector set up can be seen in Fig. 1(A) and (B). The array of detectors on the 3D printed cap can be seen in Fig. 1(C). Figure 1(D) shows for a given measurement, how the log intensity changes as a function of source-detector separation. Here there is a linear decrease in log intensity, with discrete groups of log intensity corresponding to NN1-5. This measurement was made with the reference rod inserted into the phantom, i.e no contrast was present. It should be noted that the AC amplitude was used for the ’CW’ data because the AC amplitude is less susceptible to external light contamination than the DC amplitude typically used in CW experiments. Whilst the AC and DC intensity behave similarly, they are not the exact same.

There is no exact definition of what classifies as a ’high density’ arrangement of sources and detectors, although a study by Tian et al. in 2009 [30] used various numbers of source-detector densities and found that image reconstruction quality in DOT did not improve after increasing the number of overlapping measurements for a given surface area past three, which this study uses. This study does not include measurements above NN2 (29 mm) separation, since data of NN3 and NN4 do not have sufficient signal to noise ratio, however this is in line with previous high density studies, for example White and Culver in 2010 [13] evaluated ’high density’ DOT using only up to NN2 (29 mm) measurements in their high density array.

Each measurement of a rod at a given modulation frequency was completed in three stages, a reference measurement, a contrast measurement and a repeated reference measurement. To obtain a measurement, a contrast rod was inserted into the phantom. The rod was positioned such that the contrast anomaly was positioned outside the phantom. Then, a minimum of 1000 frames (time points) were recorded, then the rod was moved such that the contrast was at the centre of the phantom (X = 0 mm, Y = 0 mm, Z = -15 mm) and a further minimum of a 1000 frames were recorded. Finally the rod was moved back to its original position to collect a minimum of another 1000 frames. This process was repeated for each contrast rod (rod 3, 5 and 7) and for each modulation frequency (78 MHz, 141 MHz, 203 MHz), so that there were nine measurements in total, each comprising of a minimum of 3000 frames of data (approximately 75 s).

In order to account for low frequency system drift, the medians of the reference measurement before and after the contrast measurement were used as the baseline measurement. Then the median of the contrast measurement was used with the baseline measurement for image reconstruction. This was done so that the change in the signal due to the contrast anomaly would not be increased or decreased by a frequency filter. For example using a high pass filter effected the change in data due to the contrast anomaly. From this processing and these measurements, tomographic reconstructions of the spatial distribution of absorption coefficient were performed as outlined in the next section.

### 2.1 Tomographic reconstruction

In the following sets of equations, ⊘ denotes the Hadamard piecewise matrix division operator, and ∘ denotes the Hadamard piecewise matrix multiplication operator, otherwise standard matrix algebra applies. All variables represent matrices, unless explicitly defined as scalar values. To perform tomographic reconstructions of the spatial distribution of absorption coefficient, a mapping function, or Jacobian ( J ) (sometimes called the sensitivity or A matrix) is used to transform changes in measured data ( ∂Y ) to changes in optical properties ( ∂X ) of the medium, which is given in Eq. (2),

(2) ∂Y=J∂X.

The explanation and background of Eq. (2) has been detailed previously [24] [31] . For this study, the measurements were calibrated [32] using modelled data from an finite element model (FEM) of the phantom. Model data was generated using the forward model of photon propagation in NIRFAST [31] . The experimental datum from the reference measurement is given by $Y_{ref}$ and from the contrast anomaly $Y_{anomaly}$ . The model data from NIRFAST is $Y_{fem}$ , with the calibration factors being scaling factors for intensity ( I ) and offset for phase ( ϕ ), $I_{SC}$ and $\phi_{OFF}$ respectively, and are given in Eq. (3) and (4),

(3) $$I_{SC}=Y_{Ifem}⊘Y_{Iref}$$

(4) $$\phi_{OFF}=Y_{\phi fem}-Y_{\phi ref}\;.$$

Using Eq. (3) and (4), the scaled changes in measured data, intensity ( $\partial Y_{I}$ ) and phase ( $\partial Y_{\phi }$ ), due to the contrast anomaly are shown in Eq. (5) and (6),

(5) $$\partial Y_{I}=log((Y_{Ianomaly}\circ I_{SC})/Y_{Ifem})$$

(6) $$\partial Y_{\phi }=(Y_{\phi anomaly}+\phi_{OFF})-Y_{\phi fem}\;.$$

From these changes in measured data ( $\partial Y_{I,\phi }$ ) and using Eq. (2), the inverse of the Jacobian can be approximated ( $J_{p}$ ) using the Moore-Penrose pseudoinverse [33] ( $J_{p}^{\#}$ ) and Tikhonov regularization to calculate the changes in optical properties of the phantom, ∂X . This is shown in Eq. (7),

(7) $$\partial X=J_{p}^{\#}\partial Y_{I,\phi }\;.$$

For CW data, the intensity data at 141 MHz is used, FD data, the intensity and phase at 141 MHz are stacked in the Y and J matrix, and for mFD data, intensity at 141 MHz is used with phase data at 78 MHz + 141 MHz + 203 MHz.

The reason why intensity data at multiple modulation frequencies was not in the mFD data type was that it has been shown that there is an appreciable increase in the sampling depth of phase data for increasing modulation frequencies, but that the change in sampling depth with intensity data is very small [26], so it was decided that only additional phases would be used in the mFD data.

The structure of the data and the Jacobians for CW and FD can be seen in Eq. (8) and for mFD in Eq. (9),

(8) $$Y_{CW}=\left[\begin{array}{c} I_{141} \end{array}\right]\;J_{CW}=\left[\begin{array}{c} J_{I141} \end{array}\right]\;Y_{FD}=\left[\begin{array}{c} I_{141}\\ \phi_{141} \end{array}\right]\;J_{FD}=\left[\begin{array}{c} J_{I141}\\ J_{\phi 141} \end{array}\right]$$

(9) $$Y_{mFD}=\left[\begin{array}{c} I_{141}\\ \phi_{78}\\ \phi_{141}\\ \phi_{203} \end{array}\right]\;J_{mFD}=\left[\begin{array}{c} J_{I141}\\ J_{\phi 78}\\ J_{\phi 141}\\ J_{\phi 203} \end{array}\right].$$

Voxel and data normalization of the Jacobian are also applied prior to inversion, to allow scaling the depth sensitivity in the image reconstruction. The regularisation parameter is used to tune the balance between smoothing and over-fitting of a solution. A normalization parameter, β (scalar) and the regularization parameter, λ (scalar) are both set to 0.01. These values are chosen empirically and have been used in prior studies [13,24].

Voxel normalization of the Jacobian is applied to each column of J, denoted by J(columns) by Eq. (10),

(10) $$J(columns)=J_{p}⊘\sqrt{L_{V}+\beta *max(L_{V})}$$

where $L_{V}=\Sigma J_{voxel}^{2}$ (sum of columns in the Jacobian and each column represents a unique voxel), and data normalization is applied to each row of J, denoted by J(rows) by Eq. (11),

(11) $$J(rows)=J_{p}⊘M$$

where $M=\sqrt{L_{D}+\beta *max(L_{D})}$ and $L_{D}=\Sigma J_{data}^{2}$ (sum of rows in the Jacobian and each row represents a unique source-detector channel). Then, Tikhonov regularisation is applied during the tomographic reconstruction which gives the change in optical properties of the phantom shown in Eq. (12),

(12) $$\partial X=J^{T}\left(\frac{\partial Y_{I\phi }⊘M}{\left(H+R\right)}\right)⊘L_{V}^{T}$$

where the superscript *^T^* denotes the matrix is transposed, J is the normalized Jacobian of the phantom, H is the Hessian which is, $H=JJ^{T}$ , R is the regularisation term,

R=⊮λ∗max(diag(H)) and ⊮ is the identity matrix. For iterative image reconstruction, Eq. (10), (11) and (12) are applied for every iteration and λ has a starting value of λ=1000 and decreases by a factor of $10^{0.2}$ at each iteration. In this work, only $\mu_{a}$ reconstructed images are shown.

**Fig. 2.**
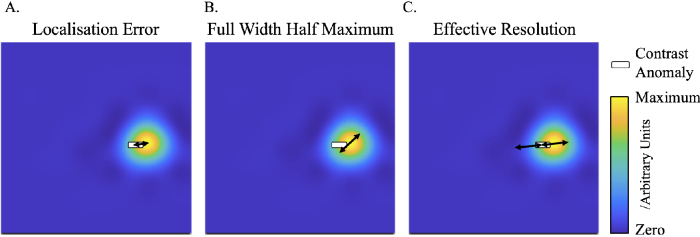
A diagram of how each performance metric [13, 24] is calculated. A. The LOCA is given by the distance between the centre of the contrast anomaly and the maximum recovery of absorption coefficient in the reconstructed image. B. The FWHM is given by the maximum distance between any two nodes that are more than or equal to 50 % of the maximum of the reconstructed image. C. The ERES is twice the maximum distance between the centre of the contrast anomaly and any node that is more than or equal to 50 % of the maximum of the reconstructed image.

To evaluate the performance of the image reconstructions, three evaluation metrics are calculated and used. These are the localisation error (LOCA), the full width half maximum error (FWHM) and the effective resolution (ERES). The metrics were chosen from their use in previous related studies [13, 24] . The LOCA is the distance between the centre of the contrast anomaly and the maximum of the absorption coefficient the reconstruction. The FWHM is the maximum distance between any two nodes where the reconstruction is above half the maximum of the reconstruction of absorption coefficient. The ERES is twice the maximum distance between the location of the contrast anomaly and any node above half the maximum value of the absorption coefficient in the reconstruction. This is visualized in Fig. 2.

To constrain the effect of artifacts in the reconstructed images, spatial constraints are applied to the four evaluation metrics. Only nodes within ± 13.8 mm in the x axis, ± 9.2 mm in the y axis and below -10 mm in the z axis in the phantom are considered for metric evaluation. The constraints in the x and y axis are half the field of view (FOV) of the source-detector placement and are outlined by solid black lines in the results. Computation was performed using a 64 bit Windows 10 PC, using Matlab R2020a and NIRFASTer [31], with an Intel i7-9700 (3.0 GHz), 32 GB RAM and a Nvidia Quadro RTX 4000 graphics card. For reference, the time taken for a single iteration, single modulation frequency forward data generation was 0.99 s, and reconstruction was 8.12 s. For single iteration multi frequency (3), the forward data generation took 1.53 s and reconstruction was 16.2 s.

## 3. Results

**Fig. 3.**
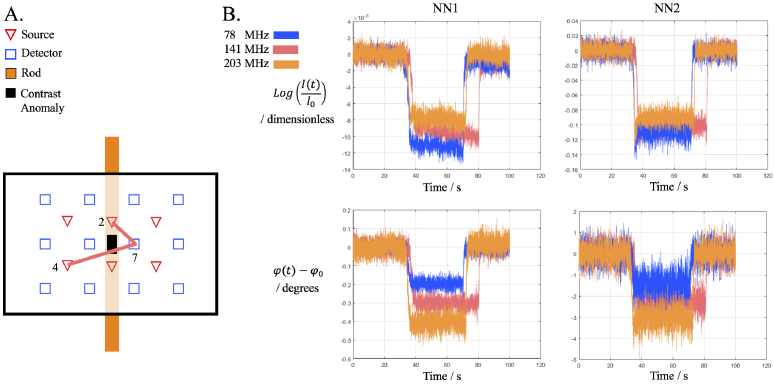
A. A schematic of which source-detector pairs are used for the data shown in Fig. 3(B). B. Log Intensity and Phase measurements of the reference rod volume and contrast anomaly of rod 7. NN1 measurement is between Source 2-Detector 7 of 13 mm separation and NN2 measurement is between Source 4-Detector 7 of 29 mm separation.

To contextualise the results of the tomographic reconstructions of absorption coefficient, typical measurements are shown in Fig. 3. They display the reference measurement, followed by the contrast anomaly being moved into the middle of the phantom and then a reference measurement. This is evident by the decrease in intensity and phase respectively. The measurements are shown at the three modulation frequencies used at 830 nm and are normalised by the mean of the reference measurement. Each modulation frequency had a separate measurement (Fig. 3(B)) , which caused small variations in the length of measurement, however a minimum number of frames (1000) were always taken for the contrast anomaly and with the median values used for data analysis.

**Fig. 4.**
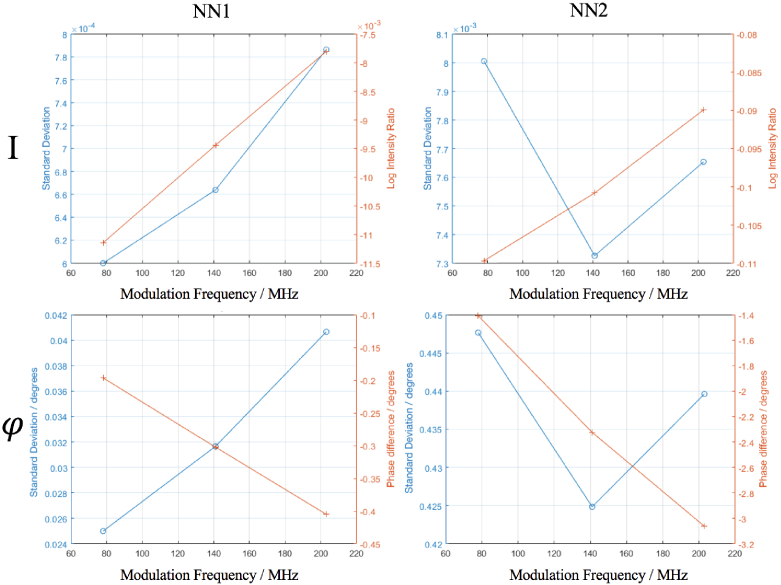
The standard deviation (left y-axis, circle ticks) and contrast (right y-axis, cross ticks) of the measurements shown in Fig. 3. The standard deviation is that of the reference measurement. For intensity the contrast is the log ratio of the mean anomaly measurement to the mean reference measurement and is dimensionless. For phase, the contrast is the difference between the mean anomaly measurement and the mean reference measurement.

**Fig. 5.**
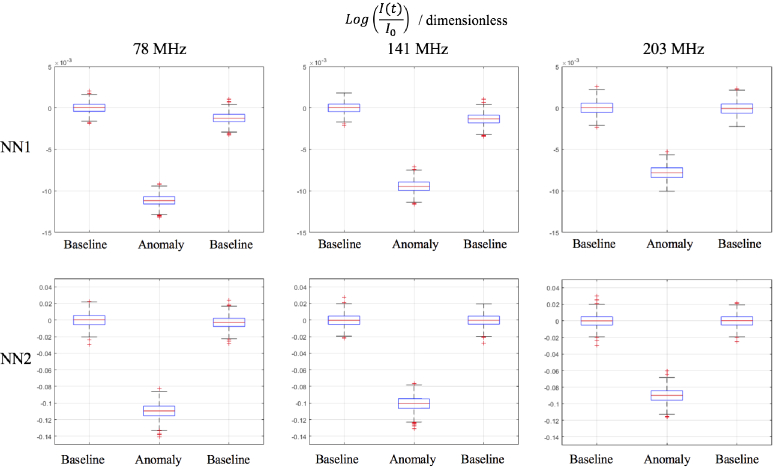
Box plots of the log ratio of intensity data of the reference rod ( $I_{0}$ ) volume and contrast anomaly ( I(t) ) of rod 7. NN1 measurement is between Source 2-Detector 7 of 13 mm separation and NN2 measurement is between Source 4-Detector 7 of 29 mm separation. Measurements are shown at the three modulation frequencies, 78 MHz, 141 MHz and 203 MHz.

**Fig. 6.**
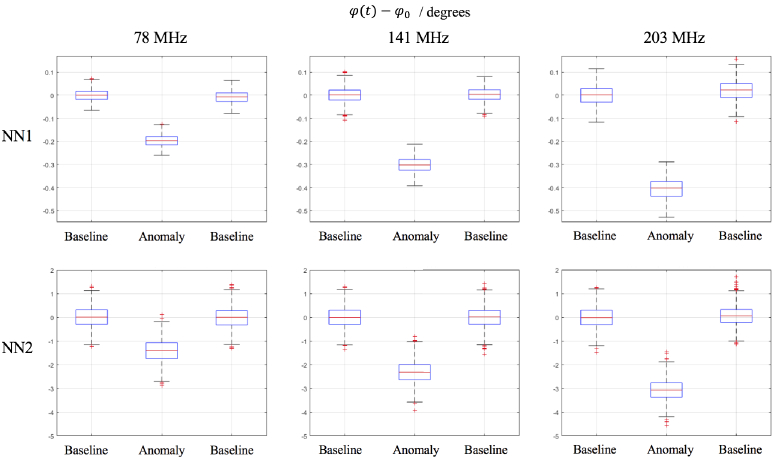
Box plots of the difference of phase data of the reference rod ( $\phi_{0}$ ) volume and contrast anomaly ( ϕ(t) ) of rod 7. NN1 measurement is between Source 2-Detector 7 of 13 mm separation and NN2 measurement is between Source 4-Detector 7 of 29 mm separation. Measurements are shown at the three modulation frequencies, 78 MHz, 141 MHz and 203 MHz.

The statistics of the data formulated in Fig. 3 are presented in Fig. 4, which shows the standard deviation of the reference measurement and the contrast induced from the contrast anomaly. Box plots of these data are shown in Fig. 5 for the intensity and Fig. 6 for the phase. For NN1 measurements, as the modulation frequency increases, the standard deviation of data increases for log intensity and phase and for NN2 the standard deviation is minimum at 141 MHz. The contrast decreases for log intensity at NN1 and NN2 as the modulation frequency increases, and for phase the opposite is true.

### 3.1 Single iteration reconstruction

**Fig. 7.**
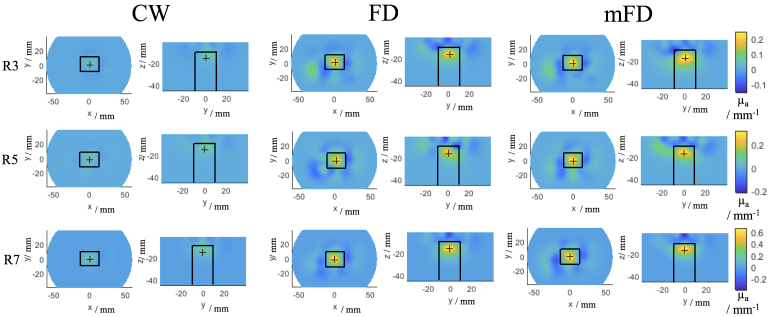
Single step tomographic reconstructions from rod 3, 5 and 7 (R3, 5 and 7 respectively) using CW, FD and mFD data respectively at 830 nm. For each reconstructed image, two views are shown, firstly the z plane at z = -15 mm and secondly the x plane at x = 0 mm. The colour bar scales are the same for each given rod. The solid black lines indicate the spatial constraints of the imaging metrics.

The tomographic image reconstructions using a single iteration are shown in Fig. 7, where each row shows contrast rod 3, 5 and 7 and each column shows the image reconstruction using CW data, FD data and mFD data. For each rod, a different colourbar scale was used but was the same for the different data types for a given rod. The first thing to note is that using NN1 and NN2 measurements, all data types reconstruct the contrast anomaly in approximately the correct location for all rods. Across all of the rods, the magnitude of the reconstructed absorption coefficient is higher using the FD and mFD data as compared to the CW data, which can be visibly seen. The maximum value of the reconstructed absorption coefficient also increases as the volume of the contrast anomaly increases, which can be seen in Table 3.

**Table 3.** A table showing the maximum values of the reconstructed absorption coefficient from the single iteration reconstructions seen in Fig. 7.

Rod N	max $\mu_{a}$ (CW) / ${\mathrm{mm}}^{-1}$	max $\mu_{a}$ (FD) / ${\mathrm{mm}}^{-1}$	max $\mu_{a}$ (mFD) / ${\mathrm{mm}}^{-1}$
3	0.078	0.227	0.254
5	0.098	0.294	0.331
7	0.227	0.653	0.689

There are artifacts present in all image reconstructions. Using the CW data, these artefacts appear to be prominent in the plane of the z axis, with a blurring in the reconstructed image seen from the surface down to the contrast anomaly. Whereas for the FD and mFD data, the artefacts in the Z direction are that of negative contrast and there is some small positive contrast blurring in the x and y directions.

The image performance metrics are shown in Table 4, which correspond to the reconstructed images shown in Fig. 7. Across all of the rods, the best LOCA is seen by using the mFD data and this general localisation performance can be observed when looking at the co-ordinates of the maximum value of absorption coefficient of the reconstructed image. Generally the maximum of the mFD and FD reconstructions are at z = - 14.5 or -15.5 mm, whereas the CW data is at z = - 11.5 mm or - 12.5 mm. The z axis error accounts for most of the differences in LOCA, however the mFD data also is more accurate in the x and y axis, with the average (x,y) co-ordinates being (0.33,1.00) mm , compared to (1.96,0.87) mm for FD and (1.30,1.89) mm for CW. The FWHM and ERES are more variable between the three data types. On average, CW data offers the best FWHM of 15.45 mm, whereas FD and mFD have an FWHM of 16.40 mm and 16.42 mm respectively. For the ERES, on average mFD (17.99 mm) performs better than FD (18.20 mm), which in turn is better than CW (19.83 mm).

**Table 4.** The evaluation metrics, the LOCA, FWHM and ERES shown for rod 3, 5 and 7 using CW, FD and mFD data respectively. These are from the reconstructed images shown in Fig. 7, using a single iteration for image reconstruction.

Rod N	Metric / mm	CW	FD	mFD
3	LOCA	4.12	2.35	**2.20**
3	FWHM	**13.81**	15.58	16.60
3	ERES	20.31	**16.82**	18.53

5	LOCA	4.51	2.35	**0.81**
5	FWHM	16.36	17.46	**15.47**
5	ERES	19.59	20.16	**17.14**

7	LOCA	3.83	2.50	**0.81**
7	FWHM	16.19	**16.18**	17.19
7	ERES	19.59	**17.64**	18.30

### 3.2 Iterative reconstruction

**Fig. 8.**
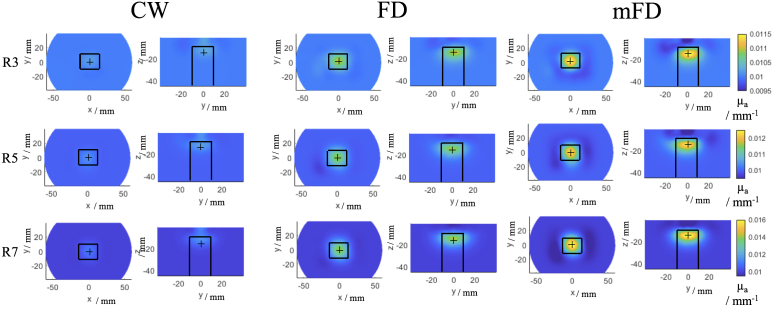
Iterative (4 iterations) tomographic reconstructions from rod 3, 5 and 7 (R3, 5 and 7 respectively) using CW, FD and mFD data respectively at 830 nm. For each reconstructed image, two views are shown, firstly the z plane at z = -15 mm and secondly the x plane at x = 0 mm. The colour bar scales are the same for each given rod. The solid black lines indicate the spatial constraints of the imaging metrics.

**Fig. 9.**
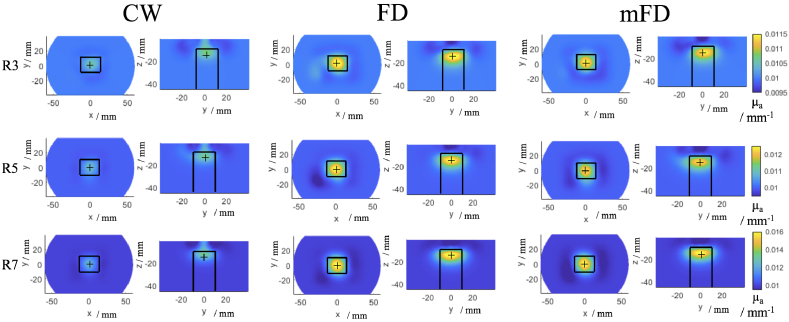
Iterative tomographic reconstructions from rod 3, 5 and 7 (R3, 5 and 7 respectively) using CW (8 iterations), FD (6 iterations) and mFD (4 iterations) data respectively at 830 nm. For each reconstructed image, two views are shown, firstly the z plane at z = -15 mm and secondly the x plane at x = 0 mm. The colour bar scales are the same for each given rod. The solid black lines indicate the spatial constraints of the imaging metrics.

The number of iterations were determined by a stopping condition, which was when each data type scored the lowest FWHM and ERES, which was the iteration before the lowest difference between the measured difference data and the forward model generated data. For CW data this was for eight iterations, for FD data this was for six iterations and for mFD data this was for four iterations. This means that the highest number of iterations and therefore the same amount of regularisation applied to each data type are four iterations, which are shown in Table 5 and Fig. 8. The results from the highest number of iterations for each data type are shown in Table 6 and Fig. 9.

**Table 5.** The evaluation metrics, the LOCA, FWHM and ERES shown for rod 3, 5 and 7 using CW, FD and mFD data respectively. These are from the reconstructed images shown in Fig. 8. This is using four iterations for each data type.

Rod N	Metric / mm	CW	FD	mFD
3	LOCA	3.83	2.33	**2.20**
3	FWHM	17.72	19.75	**13.23**
3	ERES	30.67	21.61	**15.66**

5	LOCA	3.83	**0.81**	**0.81**
5	FWHM	17.42	20.35	**12.24**
5	ERES	30.39	20.91	**13.31**

7	LOCA	3.83	**0.81**	**0.81**
7	FWHM	16.89	20.88	**14.92**
7	ERES	30.39	21.36	**15.66**

**Table 6.** The evaluation metrics, the LOCA, FWHM and ERES shown for rod 3, 5 and 7 using CW, FD and mFD data respectively. These are from the reconstructed images shown in Fig. 9. This is using a eight iterations for CW data, six iterations for FD data and four iterations for mFD data.

**Rod N**	**Metric / mm**	**CW**	**FD**	**mFD**
3	LOCA	4.12	2.32	**2.20**
3	FWHM	**13.08**	16.86	13.23
3	ERES	18.75	18.38	**15.66**

5	LOCA	3.83	**0.81**	**0.81**
5	FWHM	**11.25**	17.20	12.24
5	ERES	15.27	17.78	**13.31**

7	LOCA	3.82	**0.81**	**0.81**
7	FWHM	**11.24**	17.60	14.92
7	ERES	**15.04**	18.76	15.66

For N = 4 iterative reconstructions of absorption coefficient the images are visually smoother as compared to single step reconstructions. Each reconstructed image shown is after four iterations and the colourbars are the same for each data type for a given rod. Similar to the single step iterations in Fig. 7, the CW data shows positive artifacts in the z axis above the contrast anomaly, whereas the FD and mFD data shows negative contrast artifacts. The CW artifacts are more an extension of the anomaly itself, blurring in the z direction, whereas the FD and mFD artifact is of negative contrast and just below the surface of phantom. The magnitude of the reconstructed absorption coefficient increases as the contrast increases, which was the case for the single step reconstruction. The magnitude of reconstructed absorption coefficient is largest for mFD data, as shown by rod 3, where mFD data is 0.0115 $\mu_{a}$ / ${\mathrm{mm}}^{-1}$ , compared to 0.0110 $\mu_{a}$ / ${\mathrm{mm}}^{-1}$ for FD data and 0.0103 $\mu_{a}$ / ${\mathrm{mm}}^{-1}$ for CW data.

The imaging metrics are shown in Table 5, the LOCA is the best for FD and mFD data, where it is 0.81 mm for rod 5 and 7 respectively. mFD data has the best FWHM and ERES across all rods, with an average FWHM of 13.46 mm, compared to 20.32 mm for FD and 17.34 mm for CW data, and for the ERES, mFD data is 14.87 mm, compared to 21.29 mm for FD and 30.48 mm for CW data.

For a higher number of iterations, the magnitude of absorption coefficient in the reconstructed image increases, which is why the reconstructed image from CW data appears brighter and tighter relative to the FD and mFD data compared to Fig. 7 and 8. This increases the performance of the CW data, as it causes the artifact in the z axis to have less influence on the imaging metrics. The use of more iterations also helps the FD data, as the reconstruction of the contrast anomaly also appears brighter and is less spread out compared to the lower number of iterations shown in Fig. 7 and 8.

Table 6 shows the imaging metrics corresponding to the reconstructed images shown in Fig. 9. The LOCA remains virtually identical to that of the four iterations shown in Table 5, with the only differences being for CW and FD data for rod 3 and 7. The increased number of iterations of CW and FD data causes the average FWHM and ERES to reduce compared to four iterations, with the reductions being more significant for the CW case. For the FWHM these reductions are 17.34 mm to 11.85 mm for CW, and 20.32 mm to 17.22 mm for FD, and for the ERES the changes are 30.48 mm to 16.35 mm for CW and 21.29 mm to 18.30 mm for FD data. Overall CW data has a lower FWHM, whereas mFD data has the lowest ERES, and in both cases FD performs worse than either CW or mFD data.

## 4. Discussion

This study assesses the performance of real mFD-HD-DOT measurements on a phantom, which means the relative merits and disadvantages of mFD data are evaluated. One of the main criticisms of using phase data is how it is inherently nosier than intensity data for a given modulation frequency and that the contrast to noise ratio of phase data should increase with source-detector separation and modulation frequency [7]. This leads to questions about the inclusion of phase data, such as is the contrast in phase data in functional brain imaging sufficient to perform accurate DOT despite the noise. The experimental data shown in Fig. 3 attempts to answer the aforementioned questions, by showing intensity and phase data at three modulation frequencies (78 MHz, 141 MHz and 203 MHz) at two different source-detector separations (NN1 = 13 mm and NN2 = 29 mm). From the statistics in Fig. 4, the contrast to noise ratio is smaller in the phase measurements than intensity, and for both intensity and phase increases as the source-detector separation increases, which is already well established. There appears to be a trade off when choosing a modulation frequency and what data types to include between the noise of the signal, and the quality of information content (contrast and depth sensitivity) that can be obtained. This is demonstrated by the approximately linear and inverse relationship that the intensity and phase data have with each other with respect to their signal to noise ratio, such that the lower frequency for intensity and the higher frequency phase offer the best signal to noise ratio. The limitation of this analysis is that only NN1 and NN2 are investigated. In some ’high density’ arrangements, NN3 (39 mm) and NN4 (46 mm) can be used [5], however were excluded from use in reconstruction in this study because the signal was too low (Fig. 1(D).). This is due to the intensity of light being too strong from NN1 measurements, which causes the voltage bias of detectors to be low, and this means there is lower signal from higher NN measurements.

Overall across the three contrast rod using the single iteration reconstruction of absorption coefficient, mFD performed better than FD or CW data (Table 4). For the LOCA, mFD was the smallest error each time, followed by FD and then CW was the largest LOCA. For the FWHM and ERES metrics, the performance was variable across the three data types, however only in one instance was the smallest FWHM or ERES from CW data (rod 3), for rod 5 and 7, the smallest FWHM or ERES was from FD or mFD data. This is due to the increased magnitude of maximum reconstructed absorption coefficient from the FD and mFD data, as a more concentrated, less broader reconstruction will yield lower metrics. The ERES is closely coupled to the LOCA, with a a larger LOCA effectively scaling the ERES, which is why CW data never had the best LOCA or ERES. The FWHM is independent of these two metrics and is influenced by the tomographic reconstruction itself and is not influenced by the true location of the contrast anomaly. Due to the lower inherent noise of CW data, which should lead to a ’cleaner’ reconstructed image the FWHM for CW was comparable (rod 5 and 7) if not better (rod 3) than for FD or mFD. However, the main disadvantage of the CW data is that the sensitivity is shallower compared to FD data [14], which can be seen by the reconstructed images in Fig. 7 for all three rods and the fact that CW had a tendancy to localize the anomaly a 2-3 mm higher compared to FD or mFD. There is an upward tail to the reconstruction of the contrast anomaly which extends to the surface of the phantom from the CW case, which is not observed for the FD or mFD case. As is previously established, phase data samples deeper than intensity data [14,26] , which are evident in the FD and mFD reconstruction of absorption coefficient. However, the FD and mFD reconstructions suffer from some positive and negative contrast artifacts, which are seen above the contrast anomaly instead of the positive tail in the CW case. The spatial constraints on metrics will dampen the effect of these artifacts for all three data types, mainly the FWHM for CW and ERES for FD and mFD.

For the iterative reconstructions of absorption coefficient of the same number of iterations (4, Table 5), mFD by far performs the best having the lowest metrics for all rods and jointly lowest for the LOCA on rod 5 and 7 with FD data. The regularisation after four iterations for mFD data causes the optimum balance between over-fitting noisy data and under-fitting data and excluding features in the signal. Just like for the single iteration case, mFD data has the highest magnitude of reconstructed absorption coefficient at four iterations, which is visible in Fig. 8, which will favour the LOCA and ERES metrics. Unlike the single iteration case where for rod 3 and 7 CW had a lower FWHM than mFD, mFD has smaller FWHM’s, which could be due to under-fitting of CW data ,since CW data has it’s best solution at N = 8 iterations. Generally the artifacts present for the single iteration case appear smoothed out for the multi iteration case, particularly in the x-y direction for FD and mFD data, however the shallow tail still appears for CW and a negative contrast artifact is still present for mFD.

For the case of the best number of iterations (8-CW, 6-FD, 4-mFD, Table 6), CW performs better compared to N = 4 number of iterations. mFD and FD still perform the best for the LOCA, and the ERES is lowest from mFD for rod 3 and 5. CW data can go to a higher number of iterations compared to FD or mFD as it is less susceptible to noise, which helps refine the reconstructed image, as demonstrated by the lowest FWHM metrics for all three rods. However, this does not eliminate the inherent sampling profile of CW data, as the localisation is still the worst compared to FD or mFD. For the case of rod 7, even though the FWHM is 28.1% lower in CW compared to mFD, the poor LOCA means that the ERES is only 4.03% lower.

The reconstructed values of the absorption coefficient in the single iteration case are higher than that of the reported equivalent absorption coefficients (Table 2), which could be because an non optimal regularisation parameter for imaging the phantom was used, however the regularisation parameter chosen to be consistent with previous studies [13] [24]. For the N = 4 iterative case, the values of reconstructed absorption coefficient are smaller than the reported values in Table 2, which could be due to the higher regularisation parameter used and the use of multiple iterations. The absorption coefficients were quoted in this study to see the relative difference of them between the data types. Further study is warranted to compare these to the given values of the phantom, which could include using a higher resolution FEM of the phantom and optimising the regularisation parameter to obtain the absorption coefficients in the nodes where the contrast anomaly are.

There are four limitations of this study. Firstly, only measurements up to NN1 (13 mm) and NN2 (29.02 mm) were used, which excludes NN3 (39 mm) and NN4 (46 mm). Some HD-DOT experiments, channels above 30 mm source-detector separation are included [5], however due to the high intensity of light from NN1 channels, the voltage bias of detectors had to be lowered in order to not saturate the measurement, which meant the dynamic range was shifted away from the higher source-detector separations. However due to the nature of the study and the depth of the contrast anomaly (-15 mm), NN1 and NN2 measurements were completely sufficient to perform HD-DOT. Secondly, for measurements at different modulation frequencies, the contrast anomaly was manually moved to the centre position ( x = y = 0 mm), which means there may have been small differences between the true location of the contrast anomaly. However, these differences would be on order of 0 mm to 2 mm, which for HD-DOT reconstructing a contrast anomaly at 15 mm depth, is a lot smaller than the imaging resolution. Finally, spatial constraints were applied to the evaluation metrics after image reconstruction to dampen the effects of artifacts away from the contrast anomaly, as to measure the performance of the three data types with the benefit of prior information. These spatial constraints were chosen to be within the FOV of the source-detector probes on top of the phantom.

Finally, some of the increase in imaging performance of FD and mFD data compared to CW data could be attributed to the longer integration time of measurement (more samples used), since FD data contains CW data and mFD data contains FD data, which would increase the SNR of FD and mFD data. However, if the additional data was redundant (i.e the phase at one frequency for FD and the phase at multiple frequencies for mFD), then it would not provide a significant advantage, since the CW data is already using 2000 samples for the reference and 1000 samples for the perturbation measurements respectively. In addition, the use of FD and mFD data in this study represents realistic use cases of FD and mFD systems in real practice [27] as FD systems collect intensity and phase data simultaneously, so there would be no reason not to use all of the phase and multi-frequency data in analysis. The ISS Imagent system is the worst case scenario for mFD data, since it can only measure at one modulation frequency at a time, whereas new systems are being developed to measure multiple modulation frequencies simultaneously [27], meaning no extra time or repeated measurements are required to collect mFD data.

For future studies and experiments using FD systems and considering what modulation frequency to use and if modulation frequencies should be combined, there exists a trade-off. Figure 4 shows that for NN1 and NN2 measurements, the higher modulation frequencies used, there will be increased phase contrast, but lower intensity contrast and that these relationships are linear. For NN1 measurements, the noise of intensity and phase data increases linearly as the modulation frequency increases, however for NN2, the noise behaves like a quadratic curve, with the minima being at 141 MHz. The quadratic nature of the standard deviation could be due to the characteristics of the instrumentation, rather than something fundamental about the modulation frequency. Too high a modulation frequency may lead to an immeasurable signal, as experimentally when using the ISS Imagent, increasing the modulation frequency meant the voltage bias of detectors had to be increased, otherwise there would not be a signal, and for frequencies above 300 MHz the AC and DC signal was very weak. For the combination of modulation frequencies systems like the ISS Imagent can only measure one frequency at a time, so for N frequencies, N repeated measurements have to be taken. On a phantom this factor is easy to overcome, however for in-vivo imaging practical problems may arise, such as source-detector probes moving position between measurements and the increased time by factor N of data acquisition. For those reasons and not wanting to use redundant data as suggested by Intes et al. [17] , the three modulation frequencies used in this study represent a feasible number of frequencies to use. Finally higher modulation frequencies have a deeper distribution of sensitivity compared to lower modulation frequencies [26], which means what modulation frequency and the number of frequencies to use depends strongly on the geometry of what is being imaged, the depth sampling required, source-detector separation and any unique experimental factors, such as if repeat measurements are possible and if the system can acquire multiple frequencies simultaneously.

## 5. Conclusion

This study assesses the use of multiple modulation frequency data in frequency domain high-density diffuse optical tomography. Three data types were compared, CW (intensity at 141 MHz), FD (intensity and phase at 141 MHz) and mFD (intensity at 141 MHz and phase at 78 MHz + 141 MHz + 203 MHz), and overall mFD performed better than FD and CW data across single and iterative reconstruction of absorption coefficient. This is shown by the fact for single iteration reconstruction (Table 4), mFD scored the best metric in five out of nine cases, and for the N = 4 iterative reconstruction case (Table 5), where the same regularisation was applied to each data type, mFD scored the best metric seven out of nine times and joint best with FD in the other two cases.

Generally, FD performed better than CW data, particularly in the localisation of the contrast anomaly, however as demonstrated in the iterative reconstructions, FD reconstructed images were broader and so CW demonstrated lower FWHM. This is where the using multiple modulation frequencies was most effective, as mFD data in iterative reconstruction considerably increased the accuracy and resolution of tomography. The use of three frequencies could be considered a practical limit for typical in-vivo measurements, as to use more frequencies would require more acquisitions of data being taken, as the system used was not capable of simultaneous modulation frequencies. Future studies using FD data should consider if it feasible to collect data at multiple modulation frequencies simultaneously, and for systems capable of simultaneous frequency acquisition, then mFD data is essential to unlock the best performance from FD imaging.

## Data Availability

Data underlying the results presented in this paper are not publicly available at this time but may be obtained from the authors upon reasonable request. Please contact the lead author via email, or Twitter @BrainImagingGuy.
